# Hepatoid adenocarcinoma of the gallbladder

**DOI:** 10.1186/1477-7819-9-103

**Published:** 2011-09-13

**Authors:** Sameh Ellouze, Charfi Slim, Guirat Ahmad, Gouiaa Naourez, Amouri Ali, Mnif Héla, Kossentini Mariem, Ben Amar Mohamed, Boudawara Tahia

**Affiliations:** 1 Department of pathology, Habib Bourguiba Hospital, road El Ain., 3029, Sfax, Tunisia; 2 Department of surgery, Habib Bourguiba hospital, road El Ain., 3029, Sfax, Tunisia; 3 Department of gastroenterology, Hedi Chaker hospital, road El Ain., 3029 Sfax, Tunisia

**Keywords:** Hepatoid, adenocarcinoma, gallbladder, histopathology, liver, hepathocellular carcinoma

## Abstract

Hepatoid adenocarcinoma is a rare variant of extrahepatic adenocarcinoma which behaves like hepatocellular carcinoma in morphology and functionality.

We present a rare case of hepatoid adenocarcinoma of the gallbladder which invades deeply the liver bed, in a 59-year-old woman. Histologically, most of the mass in the gallbladder was composed of cells with eosinophilic cytoplasm arranged in a trabecular pattern, which resembled hepatocellular carcinoma. The main differential diagnosis was hepatocellular carcinoma with invasion into the gallbladder. The gallbladder origin of the hepatoid adenocarcinoma was verified by the presence of foci of conventional adenocarcinoma, the recognition of high-grade dysplasia in the adjacent epithelium and the absence of cirrhosis.

## Background

Hepatoid adenocarcinoma (HAC) is a variety of adenocarcinoma associated with hepatic differentiation. The most frequent site of this carcinoma is the stomach. Only a few cases of HAC of the gallbladder have been reported [1].

We present a rare case of HAC of the gallbladder which, to the best of our knowledge, represents the eighth reported case in the English literature.

## Case report

A 59-year-old woman presented with abdominal pain and complains about general fatigue.

**Figure 1:**
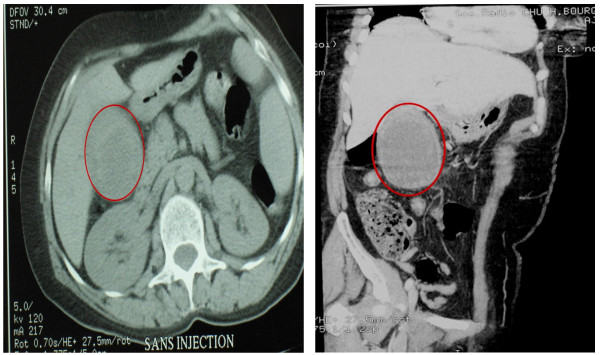
**Abdominal computed tomography revealed a solid mass within the gallbladder**. **1a and 1b: **Abdominal computed tomography revealed a solid mass within the gallbladder, and absence of liver nodules.

Ultrasonography and computed tomography revealed a solid mass within the gallbladder, which infiltrates the adjacent liver without signs of cirrhosis (Figure 1a, b). Laboratory data was within normal limits. The levels of serum AFP were not assessed preoperatively. Serological tests for hepatitis B virus surface antigen and hepatitis C virus antibody were negative.

Cholecystectomy with resection of the involved liver subsegments, and regional lymph node dissection were performed.

Gross examination revealed a white and yellow solid tumor, measuring 11 × 5 cm, occupying the body of the gallbladder and invading the liver bed.

**Figure 2:**
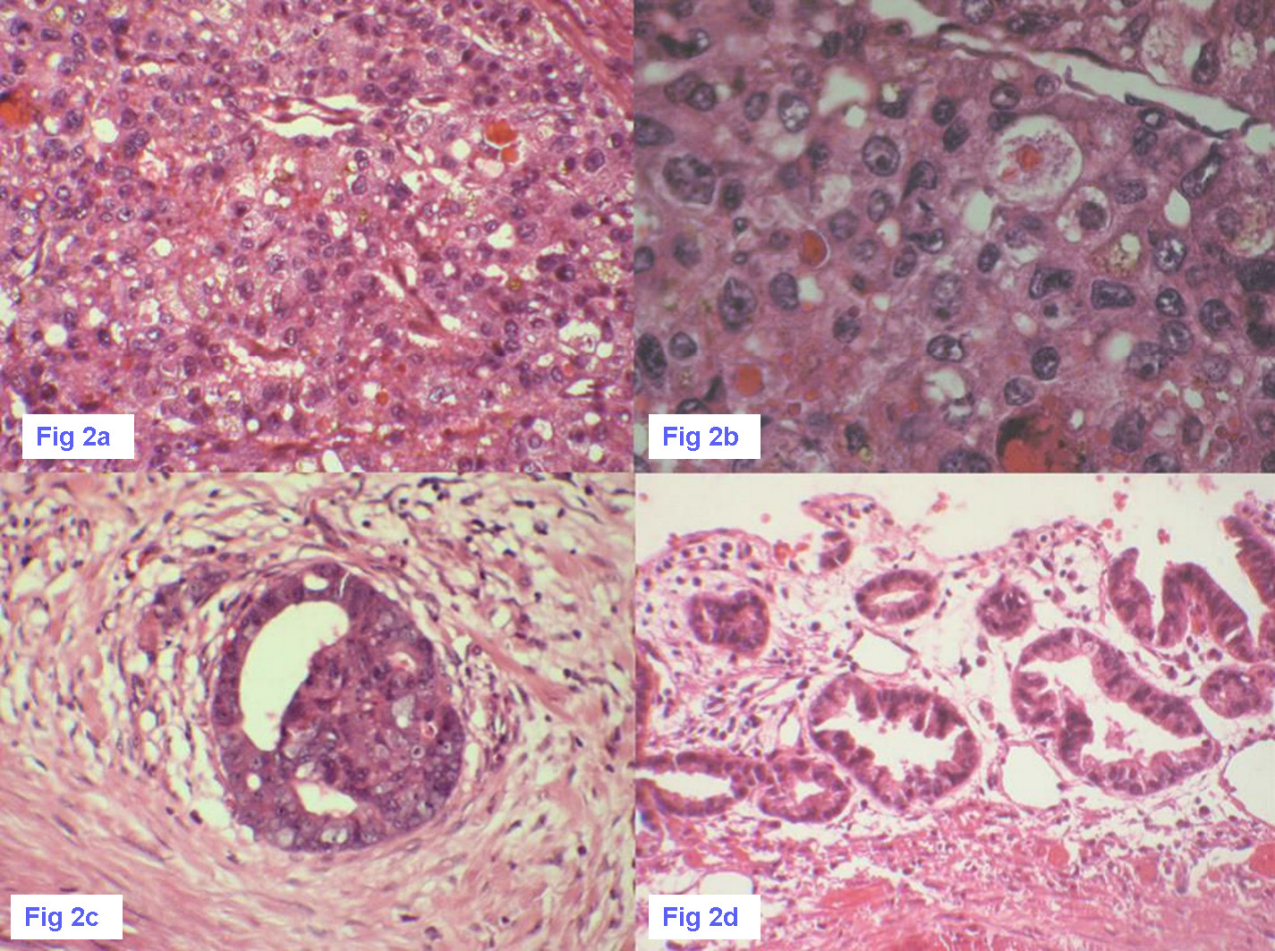
**Tumor cells are arranged in a trabecular and solid pattern**. **2a**: Tumor cells are arranged in a trabecular and solid pattern and containing bile plugs (HE×100), **2b**: Tumor cells have abundant eosinophilic cytoplasm, enlarged nuclei and prominent nucleoli (HE×400). **2c**: Foci of well-differentiated gallbladder adenocarcinoma (HE×400.), **2d**: Foci of high-grade dysplasia were observed in the gallbladder epithelium adjacent to the tumor (HE×200)

Microscopically, the tumor was composed mainly of "hepatoid cells", which were characterized by eosinophilic cytoplasm, enlarged nuclei, prominent nucleoi, and arranged in nests or proliferated in a trabecular and solid pattern. A few sporadic foci of adenocarcinoma were mixed with the hepatoid component. Bile plugs were recognized intracellularly and foci of high-grade dysplasia were observed in the gallbladder epithelium adjacent to the tumor (Figure 2). The tumor invaded deeply the liver bed. None of the lymph nodes dissected at surgery showed metastasis by the tumor cells.

**Figure 3:**
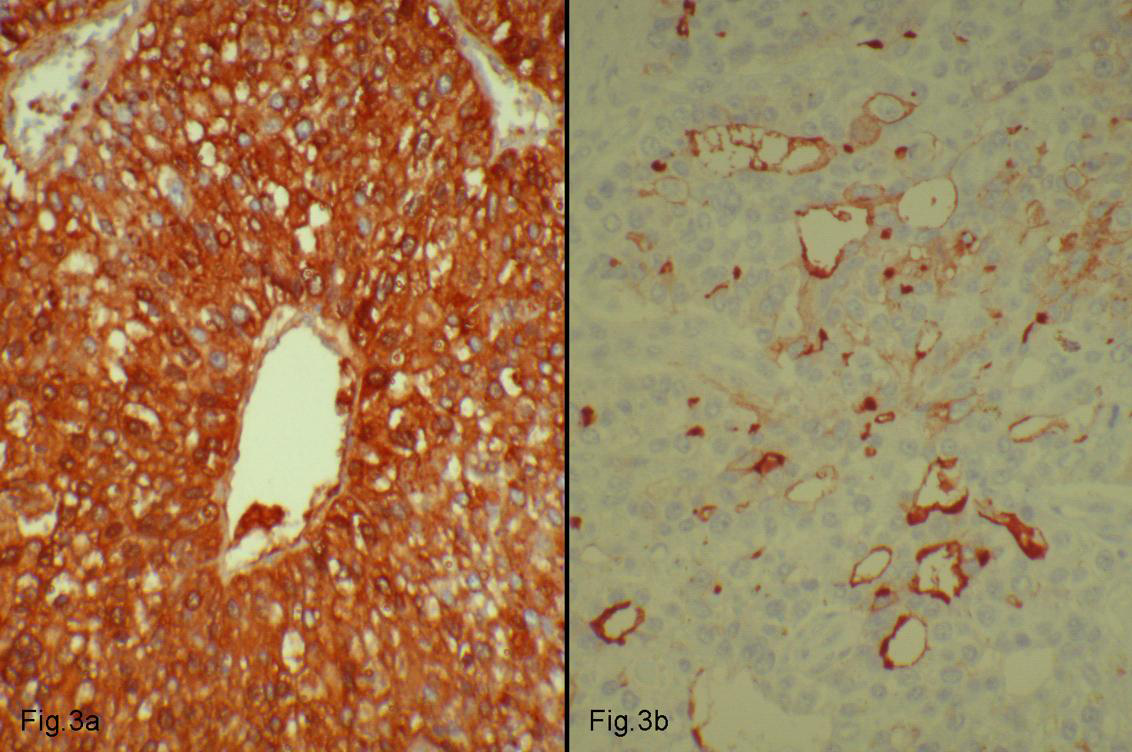
**Intracytoplasmic expression of HepPar-1**. **3a: **Intracytoplasmic expression of HepPar-1. **3b**: Canalicular staining pattern for CD10.

Immunohistochemically, the carcinoma with hepatoid features was diffusely stained for α fetoprotein (AFP), HepPar-1 (Figure 3a) and Hepatocyte-cell antibodies, but not for Keratin7, whereas the well-differentiated adenocarcinoma was immunoreactive for Keratin7 but not for AFP or Hepatocyte-cell. CD10 positivity indicated canalicular differentiation (Figure 3b).

On the basis of histologic and immunohistochemical findings, the diagnosis of HAC of the gallbladder was reported. Three months postoperatively, the patient is still alive without any further therapeutic intervention.

## Discussion

Hepatoid adenocarcinoma (HAC) is a rare variety of extrahepatic adenocarcinoma, consisting of foci of both adenomatous and hepatocellular differentiations which behave like hepatocellular carcinoma (HCC) in morphology and functionality [1].

HAC was proposed as a specific type of primary gastric cancer by Ishikura et al. in 1985 [2].

Since then, carcinomas with hepatoid differentiation have been described in a variety of anatomic locations including the lung, kidney, female reproductive tract, pancreas, and gallbladder, the stomach being the most prevalent site [2].

Typically, an elevated level of serum alpha-fetoprotein (AFP) is detected, although normal levels have also been reported [1] and clear cell carcinomas of the gallbladder with or without hepatoid differentiation are often associated with high serum levels of AFP [3]. At imaging the tumor may mimic HCC [1].

As mentioned above, HAC was named because of its characteristic histopathological features, suggesting hepatoid differentiation resembling HCC. Generally, the tumor is composed mainly of large or polygonal cells with abundant eosinophilic cytoplasm, and it proliferates in a solid or trabecular pattern, although it sometimes shows medullary proliferation [4].

Furthermore, some parts which show papillary or tubular structures are often observed in the lamina propriae and/or submucosal areas [4]. The recognition of bile production proves the hepatoid nature of cells [2].

Immunohistochemically, many liver specific proteins, including AFP, albumin, transferin, PIVKA (protein induced in the absence of vitamin K), and alpha-1-antitrypsin, have been detected in the tumor cell cytoplasm. Of them, AFP is generally considered important for the diagnosis. However, it is very important to note that AFP positivity is not necessarily diagnostic of HAC, because not all HAC are associated with AFP overproduction. Therefore, the diagnosis of HAC should be made essentially by the histological features of the tumor [1].

In addition, focal positivity with Keratin7 suggested the presence of an adenocarcinoma. CD10 positivity indicated canalicular differentiation and thus hepatocellular origin [5].

The main differential diagnosis is HCC with invasion into the gallbladder. When HAC of the gallbladder invades the liver deeply (as in our case), differential diagnosis of these two tumours can be very difficult [6]. To aid differentiation, the clinical presentation of patients is important because HCC arising in non-fibrotic liver and without risk factors, such as hepatitis virus infection, is generally rare, as is lymph node metastasis at surgery. If the intramucosal foci of adenocarcinoma are detected histologically in a surgical specimen, the gallbladder origin is confirmed [6]. In our case, the gallbladder origin of the HAC was verified by the presence of foci of conventional adenocarcinoma but also by the recognition of high-grade dysplasia in the adjacent epithelium and the absence of cirrhosis.

## Conclusion

We present a rare case of hepatoid adenocarcinoma of the gallbladder which invades deeply the liver bed, in a 59-year-old woman. The main differential diagnosis was hepatocellular carcinoma with invasion into the gallbladder. The gallbladder origin of the hepatoid adenocarcinoma was verified by the presence of foci of conventional adenocarcinoma, the recognition of high-grade dysplasia in the adjacent epithelium and the absence of cirrhosis.

## Consent

A written informed consent was obtained from the patient for publication of this case report and accompanying images. A copy of the written consent is available for review by the Editor-in-Chief of this journal.

## Competing interests

The authors declare that they have no competing interests.

## Authors' contributions

SE, SC and AG formulated the manuscript, TB and NG preparated the histological figures, AA and MBA provided the clinical history and clinical figures, HM and MK participated in the design of the study. All authors read and approved the final manuscript.
